# Survival Trends in Patients with Small Intestinal Neuroendocrine Tumours—A Cohort Study in Central Norway

**DOI:** 10.3390/cancers15133272

**Published:** 2023-06-21

**Authors:** Oddry Folkestad, Øyvind Hauso, Patricia Mjønes, Reidun Fougner, Hans H. Wasmuth, Reidar Fossmark

**Affiliations:** 1 Department of Gastrointestinal Surgery, St. Olav’s Hospital, Trondheim University Hospital, 7030 Trondheim, Norway; oddfol@siv.no; 2 Department of Gastrointestinal Surgery, Vestfold Hospital Thrust, 3103 Tønsberg, Norway; 3 Department of Gastroenterology and Hepatology, St. Olav’s Hospital, Trondheim University Hospital, 7030 Trondheim, Norway; oyvind.hauso@stolav.no (Ø.H.); patricia.mjones@stolav.no (P.M.); 4 Department of Clinical and Molecular Medicine, Faculty of Medicine and Health Sciences, Norwegian University of Science and Technology (NTNU), 7491 Trondheim, Norway; hanswasmuth@gmail.com; 5 Department of Pathology, St. Olav’s Hospital, Trondheim University Hospital, 7030 Trondheim, Norway; 6 Department of Radiology, St. Olav’s Hospital, Trondheim University Hospital, 7030 Trondheim, Norway; reidun.fougner@stolav.no

**Keywords:** neuroendocrine tumours, small intestine, clinical outcome, patient survival

## Abstract

**Simple Summary:**

Neuroendocrine tumours in the small intestine (SI-NETs) may be cured with surgery, and oncological treatment may prolong survival. The trend in survival may improve over time due to advances in surgical or oncological treatment or if the disease is diagnosed at an earlier stage. We examined 242 patients with SI-NETs diagnosed from 2005 to 2021 and compared the first (2005 to 2012) and second (2012 to 2021) halves of the cohort concerning disease severity, surgical and oncologic treatment, and long-term survival. Overall survival was longer in the second period, particularly in the first year after diagnosis. This could not be explained by SI-NETs being diagnosed at an earlier stage or major differences in oncological treatment. Several observations suggested that the surgical quality could be better in the second period, as the number of lymph nodes and primary tumours retrieved during surgery was higher.

**Abstract:**

Improved surgical resection and oncological treatment, or an earlier diagnosis may increase survival in small intestinal neuroendocrine tumours (SI-NETs), but only few studies have examined survival trends. We aimed to examine the trend in overall survival and associated factors in SI-NET patients. All patients with SI-NETs at a regional hospital from June 2005 to December 2021 (*n* = 242) were identified, and the cohort was divided in half, constituting a first period (until November 2012) and a second period (from November 2012). Disease and treatment characteristics, including European Neuroendocrine Tumour Society (ENETS) stage, surgery, oncological treatment and survival, were recorded. The majority (*n* = 205 (84.7%)) were treated surgically and surgery was considered curative in 137 (66.8%) patients. Median survival was longer in the second period (9.0 years 95% CI 6.4–11.7 in the first period vs. median not reached in the second period, *p* = 0.014), with 5-year survival rates of 63.5% and 83.5%, respectively. ENETS stage and oncological treatment did not differ between the periods, but factors associated with surgical quality, such as lymph node harvest and resection of multiple SI-NETs, were significantly higher in the second period. Age, ENETS stage, time period and tumour resection were independently associated with survival in a multivariate analysis.

## 1. Introduction

Neuroendocrine tumours (NETs) of the jejunum and ileum derive from serotonin-producing enterochromaffin cells and are referred to as small intestinal NETs (SI-NETs). In epidemiological studies based on clinical data, the incidence of SI-NETs has increased over several decades and is now in the range of 0.8–2.7/100,000 [1,2,3,4,5,6] However, in a population-based autopsy series, the prevalence was approximately 0.5% [7,8], suggesting that SI-NETs are far more common than reported in most epidemiological studies and SI-NETs may be asymptomatic without affecting life expectancy in the majority of patients. However, in a Swedish national cohort, the incidence of clinical SI-NETs doubled from 1960 to 2015, whereas the incidence in autopsies remained unchanged [2]. In contrast, the incidence of SI-NETs in Iceland remained unchanged from 1966 to 2017 [9]. Primary SI-NETs metastasize to mesenteric lymph nodes early in the disease course, and many patients also have liver metastases at the time of diagnosis [10,11]. Still, SI-NETs have an advantageous survival relative to many other cancers, with 5-year overall survival rates in the range of 61–77% [12,13,14,15,16,17]. Patients with localized, regional disease and some patients with distant metastases are considered for surgery with curative intent [18], while treatment with somatostatin analogues [19,20] and ^177^Lu-DOTATATE [21] improve survival in patients with metastatic disease in randomized controlled trials. However, only few studies have described trends in survival of SI-NET patients. In Swedish patients with SI-NETs, the 5-year survival has improved over the past six decades [2], whereas a similar trend in survival has not been found in other cohorts [9,16]. Increased survival could be explained by improvements in surgical and/or oncological treatment. Alternatively, a higher proportion of patients may in recent years have been diagnosed with early-stage disease due to widespread use of cross-sectional imaging in patients with minor abdominal symptoms. This study aimed to assess the overall survival trend and factors associated with survival in a regional cohort of patients with SI-NET.

## 2. Materials and Methods

### 2.1. Patients

Patients with SI-NETs in the jejunum or/and ileum were identified from the archives of Department of Pathology at St Olav’s Hospital by searching for the SNOMED codes T64xxx (small intestinal tumour), T65200 and M824xx (NET) from 1 June 2005 to 31 December 2021. In addition, patients in the same period with positive octreotide scintigraphy or ^68^Ga-DOTATOC positron emission tomography (PET)-CT, and an ICD-10 code of jejunoileal neoplasia (C17.1 and C17.2) and CT imaging supporting an SI-NET diagnosis were also considered to have SI-NETs and were included in further analyses. A total of 242 patients were identified, and retrospective analyses of the medical records were conducted. To compare treatment outcomes during the study period, the patients were divided into two equally sized cohorts of 121 patients; patients diagnosed before and after 5 November 2012 were divided into a first period and a second period, respectively. The analysis was approved by the Norwegian Centre for Research Data and the Regional Committee for Medical and Health Research Ethics, South-East Norway.

### 2.2. Radiological Imaging

All patients were examined using computed tomography (CT) at diagnosis, and the majority of operated patients underwent functional imaging with octreoscan or ^68^Ga-DOTATOC PET-CT pre- or postoperatively. All patients not treated surgically were examined with CT, and an octreoscan or ^68^Ga-DOTATOC PET-CT. Assumed metastatic mesenteric lymph nodes, distant abdominal lymph nodes, liver metastases and extra-abdominal metastases at baseline were noted. A preoperative SI-NET diagnosis was categorized as known in patients with histological evidence of SI-NETs (characteristic NET findings in haematoxylin–eosin-stained tissue sections, and chromogranin A and synaptophysin positivity according to immunohistochemical examination) or radiological imaging described by an expert radiologist as suggestive of SI-NETs (i.e., hypervascular tumours in the intestinal wall; hypervascular mesenterial mass with unsharp borders and desmoplastic reaction in the surroundings, often with calcification; enlarged hypervascular lymph nodes along the mesenterium; and hypervascular liver metastases).

### 2.3. Surgical and Non-Surgical Treatment

The type and method of surgical resection and whether it was an elective or acute procedure were recorded. Surgery was defined as acute if performed following acute hospital admission. The operation was categorized as “curative” if the patient was without macroscopic tumour tissue at the end of surgery. Further treatment during follow-up was recorded for all patients, including pharmacological treatment, embolization of liver metastases, peptide receptor radionuclide treatment (PRRT) and surgical removal of metastases in patients treated with tumour resection.

### 2.4. Disease Stage and Severity

Disease stage and severity at the time of diagnosis were assessed using the 8th edition TNM classification and the European Neuroendocrine Tumour Society (ENETS) staging system based on radiological imaging, surgical reports and histopathological examination. ENETS classifies stages 0-IIIa as localized disease, stage IIIb as regional disease and stage IV as distant metastatic disease. Tumour stage was considered localized when a tumour was confined to the bowel wall; regional when metastases to locoregional lymph nodes either on pathological reports or radiological imaging were present; and distant disease when distant metastases to other organs, peritoneal spread, retroperitoneal spread or metastases to distant lymph nodes were present. Carcinoid heart disease diagnosed using echocardiography was recorded. Proliferation rates were estimated according to the Ki-67 index in the histopathological reports and classified according to World Health Organization (WHO) grade (G1–G3).

### 2.5. Survival and Disease Recurrence

The patients were followed until death or date of censorship, with a median observation time after diagnosis of 9.4 (1.0–17.2) years and time to event of 6.2 years (0.02–17.2). CT scans were performed at intervals of 6–24 months, increasing with time after surgery according to evolving ENETS guidelines [18,22] in patients operated with curative intent, if disease recurrence was suspected during follow-up or to evaluate treatment response. Patients who were not surgically resected were followed up with CT scans every 6–12 months depending on tumour load and rate of progression. Deaths were categorized as attributable to SI-NETs or not based on information in the medical record about the clinical course of the SI-NET and other disease. Patients who died of metastasis from SI-NET, tumour compression, carcinoid heart disease, acute bowel obstruction or other conditions attributable to advanced SI-NET disease were listed as SI-NET related. In patients with insufficient information about the cause of death, it was categorized as unknown.

### 2.6. Statistical Analyses

Descriptive data are presented as frequencies (*n* (%)) or medians (range). Comparisons of overall survival between groups were analysed with the Kaplan–Meier method with the log-rank test. Variables considered to potentially influence survival were included in univariate and multivariate analyses using the Cox proportional hazards model. Hazards ratios (HRs) with 95% confidence intervals (CIs) are presented. Fisher’s exact test was used for comparisons of categorical variables between groups. Two-sided Mann–Whitney test was used for comparisons of numerical variables between groups. *p*-Values < 0.05 were considered statistically significant. All statistical analyses were conducted using IBM SPSS Statistics, version 28.0 (IBM Corporation, Armonk, NY, USA).

## 3. Results

### 3.1. Patient Demographics

The cohort of 242 patients consisted of 135 (55.8%) men and 107 (44.2%) women (Table 1). Median age at surgery or diagnosis was 70.0 years (17–91). Tumour resection was performed in 205 (84.8%) patients with median age 68.0 years (17–91), and 117 (57.1%) patients were male. The 37 patients who did not undergo tumour resection were older than the resected patients (76.0 years versus 68.0 years, *p* = 0.01). Twenty-two patients (9.1%) did not have a histological diagnosis, eighteen in the first period and four patients in the second period, *p* = 0.003.

### 3.2. Surgical Procedures

Laparotomy with resection that included the primary tumour was performed in 205 patients. The standard surgical approach for removal of the primary tumour and regional metastases (locoregional resective surgery) was a bowel resection combined with extensive mesenteric dissection for the removal of mesenteric lymph node metastases. The most frequently performed procedures were resection of the small intestine (*n* = 100, 48.8%), right hemicolectomy with or without an extended small bowel resection (*n* = 69, 33.7%) and ileocolic resection (*n* = 26, 12.7%). Smaller proportions of patients underwent wedge resection of the small bowel without mesenteric lymph node dissection (*n* = 5, 2.4%), combined subtotal colectomy and resection of the small intestine (*n* = 2, 1%). Three patients (1.5%) were operated with multiple resections that included colonic resection, small bowel resection and resection of other adjacent locally invaded organs. Four patients (2.0%) underwent liver resections with curative intent; three patients (1.5%) had liver resections performed at the same time of the SI-NET operation, while one patient had planned liver resection performed as a stage-two surgery. The type of resection between the two periods did not differ; in the first period, 50% (*n* = 52) had segmental resections of the small bowel vs. 47.5% (*n* = 48) in the second period, while 46.2% (*n* = 46.2%) had a more extensive resection with a combination of colon and small bowel resection vs. 51.5% (*n* = 52), respectively (*p* = 0.354). A total of 137 patients (66.8%) were considered curatively operated, and the proportion was higher in the second period than in the first period (74.3% vs. 59.6%, *p* = 0.027). The majority of the operations were elective (*n* = 153, 74.6%), and the proportions did not differ between the first and second periods (Table 2). SI-NET disease was known preoperatively in 154 patients (75.1%), and this proportion tended to be higher in the second period (70.2% vs. 80.2%, *p* = 0.108). Among the 205 SI-NET resections, 157 (76.6%) were R0 resections, and the proportion did not differ between the first period and the second period (74.8% vs. 79.2%, *p* = 0.508). Multifocal tumours were found in 63 of the 205 (30.7%) patients who were treated surgically with tumour resection. Resection of multifocal tumours were more frequent in the second period than in the first period (37.6% vs. 24.0%, *p* = 0.049), which was explained by a higher proportion of multifocal tumours being resected during elective surgery (42.9% vs. 21.1%, *p* = 0.005), but not during emergency surgery (20.8% vs. 32.1%, *p* = 0.532) (Table 3). A median of eight lymph nodes were retrieved in the whole cohort for 205 patients who underwent SI-NET resection, while a higher number of lymph nodes were retrieved in the second period vs. the first period (10 vs. 5 lymph nodes, *p* = 0.037). The proportion of patients in which ≥eight lymph nodes were retrieved during elective surgery was significantly higher in the second period vs. the first period (66.2% vs. 45.3%, *p* = 0.014), but these proportions did not differ in emergency procedures (46.4% vs. 41.7%, *p* = 0.785).

### 3.3. Disease Stage and Severity at Time of Diagnosis

Disease characteristics are presented in Table 2. ENETS disease stage did not differ between the first period and the second period (*p* = 0.171). However, eleven (4.5%) patients had carcinoid heart disease, with nine patients (7.4%) in the first period versus two (1.7%) patients in the second period (*p* = 0.031). The WHO grade was known for 220 patients (90.9%), and a higher proportion of patients had G2 tumours in the second period (*n* = 55, 45.5%) vs. first period (*n* = 34, 28.1%), *p* = 0.039.

### 3.4. Surgery with Curative Intention and Disease Recurrence

A total of 137 (66.8%) patients had curative intent surgery. Forty-two (30.7%) of these patients had recurrence after curative intent surgery after a median follow-up time of 5.3 years (0.02–17.2) The recurrence was found in the liver alone in 21 (50%) of the 42 patients, whereas 14 (33.3%) had recurrences in both the liver and other organs. Recurrence was found in mesenteric lymph nodes alone in three (7.2%) patients, in retrocrural or retroperitoneal lymph nodes in one (2.4%) patient and in mesenteric lymph nodes and organs other than the liver in one (2.4%) patient, and a new primary tumour in the small intestine was found in two (4.8%) patients. Operation for recurrence was performed in 13 of the 42 (31%) patients, where 5 underwent liver resections and 8 were operated due to acute bowel obstruction. The rate of curative intent resection did not differ between patients operated with elective vs. emergency procedures (66.7% vs. 67.3%, *p* = 0.932). Median recurrence-free survival was 9.5 years (95% CI 8.4–10.6). Median recurrence-free survival was 11.1 years (95% CI 8.6–13.5) for G1 tumours vs. 5.3 years (95% CI 3.9–6.8) for G2 tumours (*p* = 0.016), while five-year recurrence-free survival rates were 68.8% for G1 tumours vs. 54.7% for patients with G2 tumours.

### 3.5. Non-Surgical Treatment during Follow-Up

Non-surgical treatment during follow-up is presented in Table 2. Treatment with a long-acting somatostatin analogue was given to 126 (52.1%) patients in the entire cohort, with 52.9% in the first period versus 51.2% in the second period (*p* = 0.898). Interferon treatment was given to fourteen (5.8%) patients, with eleven (9.1%) patients in the first period and three (2.5%) patients in the second period (*p* = 0.05). Everolimus was given to twelve (5%) patients, with four (3.3%) patients in the first period and eight (6.6%) in the second period (*p* = 0.375). Nine (3.7%) patients received treatment with systemic chemotherapy, with five (4.1%) in the first period and four (3.3%) patients in the second period (*p* = 1.0). Twenty-seven (11.2%) patients received treatment with PRRT, with fourteen (11.6%) in the first period and thirteen (10.7%) in the second period (*p* = 1.0). Ten (4.1%) patients were treated with transarterial embolization (TAE) of liver metastases, with seven (5.8%) in the first period and three (2.5%) in the second period (*p* = 0.33).

### 3.6. Patients Not Resected for SI-NETs

No tumour resection was performed in 37 (15.3%) patients. In six patients, an inoperable tumour was found during laparotomy; in these patients, a biopsy was collected, and an internal bypass or diverting stoma was created in situations with acute bowel obstruction. The decision for non-surgical treatment was due to high age/serious comorbidity in 7 (18.9%) patients; tumour was assessed to be inoperable in 21 (56.8%) patients; in total, 4 (10.8%) patients declined surgery; and 3 (8.1%) patients were not assessed by a surgeon or multidisciplinary team. The distribution of localized, regional and distant disease stages was 3.1%, 32.4% and 59.5%, respectively. Median estimated survival was 4.8 years (95% CI 2.5–7.2) for this cohort of patients, while for regional and distant stages the estimated survival rates were 6.5 years (95% CI 0.4–12.5) and 4.4 years (95% CI 2.9–6.0); *p* = 0.518. By the time of censorship, with a median follow-up time since diagnosis of 7.5 years (3.4–16.9), 25 (67.6%) patients were dead. Fourteen (37.8%) died of SI-NET disease; three patients (8.1%) died of other cancer; three (8.1%) died of diseases other than cancer; and for five (13.5%), the cause of death is unknown. Treatment with a long-acting somatostatin analogue was given to 81.1%, while 13.5% were treated with PRRT; 10.8% with mTOR-inhibitor; and 2.7% with interferon. Finally, 5.4% received treatment with chemotherapy.

### 3.7. Overall Survival and Cause of Death

**Figure 1:**
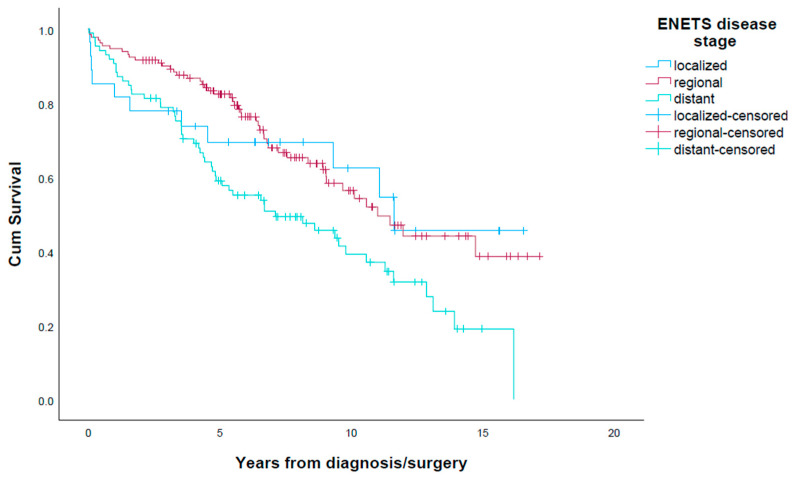
Survival in 242 patients with SI-NET stratified according to ENETS disease stage.

Amongst the 242 patients, 112 (46.3%) died after a median time of 6.2 years (0.02–17.1). Five (2.1%) patients died within a few days after surgery due to surgical complications. The 30-day and 90-day mortality rates after surgery were 2.4% and 3.7%, respectively. Fifty (20.7%) patients died due to SI-NET disease. Twenty-three (9.5%) patients had synchronous cancer, of which sixteen had colorectal cancer and twenty (8.3%) died from the synchronous cancer. Thirteen (5.4%) patients died of non-malignant disease, and the cause of death was unknown in twenty-four (9.9%) patients. Median estimated survival of the whole cohort was 10.1 years (95% CI 8.5–11.7), and 5-year survival was 73.6%. Median overall survival rates for localized, regional and distant disease stage were 10.6 years (mean 95% CI 7.9–13.2), 11.0 years (95% CI 8.8–13.2) and 7.1 years (3.4–10.8) (*p* = 0.005), with corresponding 5-year survival rates of 70.4%, 83.1% and 60.0%, respectively (Figure 1). Survival in patients with localized SI-NET was influenced by synchronous intra-abdominal cancer in 7 of the 27 (25.9%) patients, as 5 (18.5%) patients died of the synchronous cancer. Patients with G1 tumours had a median survival of 11.6 years (95% CI 8.8–14.4) vs. 8.9 years (95% CI 6.6–11.3) in patients with G2 tumours (*p* = 0.046). When comparing patients with elective surgery vs. emergency surgery for SI-NETs (*n* = 205), the median survival of the elective group was 12.0 years (95% CI 8.8–15.0) vs. 9.7 years (95% CI 7.1–12.2) (*p* = 0.136). Patients with a preoperatively known SI-NET diagnosis had a median survival of 12.0 years (95% CI 9.2–14.8), while median survival of patients with preoperatively unknown SI-NET was 9.7 years (95% CI 7.8–11.5) (*p* = 0.236). Median survival of patients who were considered tumour free after operation was 12.0 years (95% CI 8.4–15.5), compared with 8.1 years (95% CI 4.8–11.5) (*p* < 0.001) in those with residual tumour load.

### 3.8. Survival in First Period vs. Second Period

**Figure 2:**
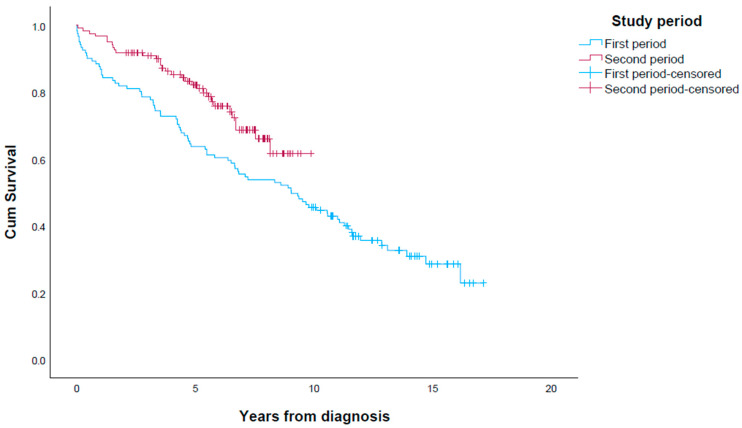
Survival in patients with SI-NET in periods 2005–2012 (first period) and 2012–2021 (second period), with 121 patients in each period.

The median survival was significantly longer in the second period (9.0 years (95% CI 6.4–11.7)) than in the first period vs. not reached for the second period (Figure 2; *p* = 0.014). The Kaplan–Meier curves of the periods separated the first year after diagnosis, and the distance persisted throughout the observation period, with 5-year survival rates of 63.5% and 83.5%, respectively. The median survival of resected patients was 9.6 years (95% CI 8.4–10.9) in the first period vs. not reached in the second period (*p* = 0.008), with 5-year survival rates of 67.3% and 88.1%, respectively.

The proportion of patients considered tumour free after resection was higher in the second period (59.6% in the first period vs. 74.3% in the second period, *p* = 0.027). Median survival of patients operated in an elective setting was longer in the second period, with *p* = 0.013, while there was no difference in overall survival after emergency surgery between the two periods (*p* = 0.34). There were numerically fewer deaths due to both surgical complications, SI-NETs, and other cancers, but there were a low number of patients, and there were no significant differences (Table 2). Patients with preoperatively known SI-NET were more often considered tumour free after elective surgery in the second period, with 70.4% vs. 52% in the first period (*p* = 0.021). However, median overall survival of patients who had curative surgery did not differ between the time periods. In a Cox proportional hazard regression model, age at surgery or diagnosis, ENETS disease stage, tumour resection and diagnosis before November 2012 were independently associated with overall survival (Table 4). 

To assess the effect of non-radical resection, we compared survival in patients who underwent R1/R2 resection (*n* = 48) vs. no resection (*n* = 37) with a Kaplan–Meier analysis and found a difference in median survival (9.0 months vs. 4.8 months, *p* = 0.035). However, due to the higher age and more advanced ENETS disease stage in patients not resected, we performed a multivariate Cox proportional hazard regression analysis that included age at surgery or diagnosis, ENETS disease stage, WHO grade and sex (Table 5). In this analysis, R1 or R2 resection was not independently associated with survival.

## 4. Discussion

### 4.1. Identifying SI-NET Patients

The incidence of SI-NETs has been reported in several registries over the past decades. A varying proportion of SI-NET patients do not have a histological diagnosis, and registries based on a histopathological diagnosis alone, such as ICD-O, thus underestimate the incidence of clinical SI-NETs [17,23]. In the current study, we found that 9% of SI-NET patients overall did not have a histological diagnosis, and the proportion was lower in the second time period. The incidence, as well as survival rates, are affected by how patients forming a cohort are identified. The inclusion of SI-NETs diagnosed at autopsy influences incidence data, but this proportion may have declined as a consequence of falling autopsy rates [2]. On the other hand, the increasing use and quality of cross-sectional imaging may have led to an increase in incidentally diagnosed SI-NETs. E.g., Stensbøl reported that the number of CT and MRI scans almost doubled from 2010 to 2020 in Sweden; in addition, higher awareness among radiologist and improved CT technology might be a contributing factor to the increased incidence of GEP NETs [5]. The above-mentioned differences in diagnostic criteria may in turn cause patient selection and explain some of the differences in reported survival rates.

### 4.2. Survival

The median overall survival in our SI-NET patient cohort was 10.1 years, which was comparable to survival in a national cohort from Iceland (9.1 years) [9] and referral centre cohort from Norway (9.3 years) [16] but considerably higher than that reported in a recent analysis of SEER data from the United States of America (52 months) [15]. Several factors were associated with overall survival. As expected, ENETS disease stage and tumour grade were associated with survival in a multivariable analysis, and these factors have been used for risk stratification of patients in routine practice [18]. The survival of patients with localized SI-NETs was relatively poor in the first five years, as many of these patients had synchronous cancer, which influenced overall survival. Resection of the primary tumour was associated with longer survival also when adjusting for age, disease stage and tumour grade. Our dataset did not allow adjusting for comorbidity or performance status that could affect patient selection, but other recent studies using propensity score matching [24] or comparison of similar cohorts [25] have reported conflicting results concerning the effect of surgical resections on overall survival in metastatic disease. Although the biological rationale [26], as well as the survival benefit [27] of resection of liver metastases has been questioned, five percent of patients in our cohort underwent liver resection, and such operations were performed in both time periods in our cohort.

### 4.3. Survival Trend

While several large studies describe trends in survival of NET patients in general [1,23], only few studies describe time trends in survival of SI-NET patients [2,9]. In the current study, we observed longer survival in the most recent period, 2012–2021, compared with the first time period, 2005–2012. In a national Swedish cohort, the survival of SI-NET patients has improved over the past six decades [2], whereas a similar trend in survival has not been found in cohorts from Iceland and Eastern Norway [9,16]. In our patient cohort, the survival curves separated the first year after diagnosis and maintained a similar distance throughout the observation period, and further analyses were performed to identify possible explanatory factors. ENETS disease stage did not differ significantly between time periods, and the difference in survival could thus not be explained by a diagnosis in earlier stages during recent years leading to improved overall survival (lead time bias) [9]. Similarly, a recent Danish study found an increase in the incidence of SI-NETs over the past decade, whereas the proportion of patients diagnosed with disseminated disease and SI-NETs diagnosed during surgery for other disease did not significantly change during the study period [5]. However, we found that fewer patients in the second time period had carcinoid heart disease, a marker of disease severity that is known to negatively affect survival [16,28]. Although evidence that both somatostatin analogues [19,20] and PRRT [21] increase survival has improved during the study period, there were no major differences between the two time periods in proportions of patients receiving the various treatments. Following clinical practice similar to that in other Scandinavian countries, many patients in our cohort were referred to PRRT many years before a survival benefit was documented in a clinical trial, which explains the lack of difference between the first and second time periods in the proportion of patients receiving PRRT. The proportions of SI-NET patients who underwent surgery did not differ between time periods. However, the overall survival of patients who underwent surgery was higher in the second period. Interestingly, specific aspects of the surgical treatment differed between time periods, suggesting that the quality of surgical treatment was better in the second period. Multiple primary tumours were found in a higher proportion of resected patients, indicating better imaging, as well as more careful exploration during the operations performed, in the second time period. The frequency of multifocal tumours was 30.7% in the present study, and this was comparable to those in other reports of patients with clinical SI-NETs, which are in the range 10.1–38.5% [9,29,30,31]. While there was no significant association between multifocal tumours, and survival or recurrence-free survival in the current study, other authors have found multifocality to be a significant negative prognostic factor of recurrence-free survival [30,32]. We found that lymph node harvest was higher in the second time period, in terms of both the median number and the proportion of patients where ≥eight lymph nodes were retrieved. There are several reports suggesting survival after SI-NET surgery to be associated with lymph node harvest [33]. A study by Landry et al. found that patients with more than seven lymph node removed experienced better cancer-specific survival than those with seven or less lymph nodes removed [34]. In an analysis of more than 11,000 SI-NET patients in the US National Cancer Database, retrieval of ≥eight lymph nodes was independently associated with survival [35]. The proportion of patients who underwent elective surgery did not change, but a higher proportion of patients were assessed as being tumour free after elective surgery in the second time period. Furthermore, there was a marked difference in overall mortality in the first year after diagnosis between the two periods. Sub-analyses with comparisons of death cause between time periods were limited by low numbers in each category. Nevertheless, we observed numerically lower numbers of deaths due to surgical complications as well as SI-NETs per se.

There has been considerable interest in whether non-curative resection, which may include removing a primary NET in patients with advanced disease stage [25,33,36], or other debulking may influence survival. In the current study, we found that although any tumour resection was independently associated with survival in a Cox multivariable analysis, there was no difference between R1/R2 resection and no resection in a Cox multivariate analysis adjusting for age and ENETS disease stage.

### 4.4. Strengths and Limitations

Assessment and treatment of SI-NETs is centralized to one hospital in Central Norway, and we are near a complete regional cohort of SI-NET patients with and without a histological diagnosis. The follow-up until death or date of censorship was complete. However, patients that were eligible neither for surgical nor for medical treatment and were thus not biopsied or examined using somatostatin receptor-based imaging could not be included in our cohort. The retrospective nature of the study and the inherent lack of randomisation of the various treatment modalities prevented causal relationships to be proven. Furthermore, the study had a limited number of patients, which restricted the possibility of performing sub-analyses. We did not have reliable data on the duration of SSA treatment for all patients, and duration was not included as a variable.

## 5. Conclusions

The survival of patients with SI-NETs improved over the study period, and this seemed related to lower mortality in the first year after diagnosis. Disease stage or oncological treatment did not change, but several factors associated with surgical quality improved during the study period.

## Figures and Tables

**Table 1:** Patient demographics, disease characteristics and treatment during follow-up in patients with SI-NETs.

	Surgical Resection of Primary SI-NET (*n* = 205)	Patients with SI-NETs Not Resected (*n* = 37)	*p*-Value
Age, median (range)	68.0 (17–91)	76.0 (52–87)	0.01
Male, *n* (%)	117 (57.1%)	18 (48.6%)	0.25
Preoperative SI-NET diagnoses, *n* (%)			
Known	154 (75.1%)		
Unknown, *n* (%)	51 (24.9%)		
Reason for no resection, *n* (%)			
Age/comorbidity		7 (18.9%)	
Patient declined surgery		4 (10.8%)	
Tumour assessed inoperable		21 (56.8%)	
Surgery not considered		3 (8.1%)	
Other cause		1 (2.7%)	
ENETS stage, *n* (%)			0.003
Localized	24 (11.7%)	3 (8.1%)	
Regional	118 (57.6%)	12 (32.4%)	
Distant	63 (30.7%)	22 (59.5%)	
Multifocal primary tumours, *n* (%)	63 (30.7%)		
WHO grade *, *n* (%)			0.168 0.20
G1	116 (56.6%)	15 (40.5%)	
G2	79 (38.5%)	10 (27%)	
Unknown	10 (4.9%)	12 (32.4%)	
Carcinoid heart disease, *n* (%)	8 (3.9%)	3 (8.1%)	
Synchronous cancer, *n* (%)	23 (11.2%)	3 (8.1%)	
Curative surgery, *n* (%)	137 (66.8%)	-	
Recurrence, *n* (%)	42 (20.5%)	-	
Deaths, *n* (%)	87 (42.4%)	25 (67.6%)	
Due to surg. complication	5 (2.4%)	-	
30-day mortality postop.	5 (2.4%)	-	
90-day mortality postop.	9 (4.3%)	-	
Due to SI-NET disease	36 (17.6%)	14 (37.8%)	
Other cancer	17 (8.3%)	3 (8.1%)	
Other disease	10 (4.9%)	3 (8.1%)	
Unknown	19 (9.3%)	5 (13.5%)	
Non-surgical treatment, *n* (%)			
SSA	96 (46.8%)	30 (81.1%)	
Interferon	13 (6.3%)	1 (2.7%)	
mTOR inhibitor	8 (3.9%)	4 (10.8%)	
TAE	10 (4.9%)	0	
Cytostatic treatment	7 (3.4%)	2 (5.4%)	
PRRT	22 (10.7%)	5 (13.5%)	
Liver surgery	12 (5.9%)	0	

WHO: World Health Organization; G: grade; Preop.: preoperatively; SI-NET: small intestinal neuroendocrine tumour; ENETS: European Neuroendocrine Tumour Society; SSA: somatostatin analogue; PRRT: peptide receptor radionuclide treatment; mTOR: mammalian target of rapamycin; TAE: transarterial embolization; * WHO grade available for 220 patients.

**Table 2:** Demographics and disease characteristics of patients with SI-NETs compared between time periods 2005–2012 (first period) and 2012–2021 (second period).

	Total (*n* = 242)	First Period (*n* = 121)	Second Period (*n* = 121)	*p*-Value
Age (median, range)		69.0	71.0	0.506
Sex (male)		70 (57.9%)	65 (53.7%)	0.605
WHO grade *, *n* (%)				0.039
G1	131 (54.1)	69 (57.0)	62 (51.2)	
G2	89 (36.8)	34 (28.1)	55 (45.5)	
Unknown	22 (9.1)	18 (14.9)	4 (3.3)	
ENETS stage, *n* (%)				0.171
Localized	27 (11.2)	14 (11.6)	13 (10.7)	
Regional	130 (53.7)	58 (47.9)	72 (59.5)	
Distant	85 (35.1)	49 (40.5)	36 (29.8)	
Carcinoid heart disease, *n* (%)	11 (4.5)	9 (7.4)	2 (1.7)	0.031
Any surgery, *n* (%)	211 (87.2)	110 (90.0)	101 (83.5)	0.123
Primary tumour resected **, *n* (%)	205 (84.7)	104 (86.0)	101 (83.5)	0.721
Preop. known SI-NET, *n* (%)	154 (75.1)	73 (70.2)	81 (80.2)	0.108
Curative surgery, *n* (%)	137 (66.8)	62 (59.6)	75 (74.3)	0.027
Lymph nodes in specimen, median (range)	8 (0–62)	5 (0–30)	10 (0–62)	0.037
Synchronous cancer in specimen	23 (11.2)	12 (11.5)	11 (10.9)	1.0
Relapse/death in radically resected patient	72 (52.6%)	42 (40.4%)	30 (29.7%)	0.09
Liver surgery, *n* (%)	12 (5.0)	4 (3.3%)	8 (6.6%)	0.247
Non-surgical treatment				
SSA		64 (52.9)	62 (51.2)	0.898
Everolimus		4 (3.3)	8 (6.6)	0.375
PRRT		14 (11.6)	13 (10.7)	1.0
Interferon		11 (9.1)	3 (2.5)	0.050
Chemotherapy		5 (4.1)	4 (3.3)	1.0
TAE		7 (5.8)	3 (2.5)	0.33
One-year mortality, *n* (%)	21 (8.7)	17 (14)	4 (3.3)	0.005
Surg. complication	5 (2.1)	4 (3.3)	1 (0.8)	0.37
SI-NET	5 (2.1)	4 (3.3)	1 (0.8)	0.37
Other cancer	4 (1.7)	3 (2.5)	1 (0.8)	0.62
Other disease	3 (1.2)	2 (1.7)	1 (0.8)	1.0
Unknown cause	4 (1.7)	4 (3.3)	0	0.12

WHO: World Health Organization; G: grade; Preop.: preoperatively; SI-NET: small intestinal neuroendocrine tumour; ENETS: European Neuroendocrine Tumour Society; SSA: somatostatin analogue; PRRT: peptide receptor radionuclide treatment; TAE: transarterial embolization. * WHO grade available for 220 patients. ** For 205 patients who underwent resection of primary tumour.

**Table 3:** Tumour pathology in patients resected for SI-NETs compared between time periods 2005–2012 (first period) and 2012–2021 (second period). LN: lymph nodes; * first period vs. second period.

	Total (*n* = 205)	First Period (*n* = 104)		Second Period (*n* = 101)		*p*-Value *
		Elective (*n* = 76)	Emergency (*n* = 28)	Elective (*n* = 77)	Emergency (*n* = 24)	
T stage, *n* (%)						0.277
0	2 (1.0)	1 (1.3)	0	1 (1.3)	0	
1	12 (5.9)	5 (6.6)	3 (10.7)	2 (2.6)	2 (8.3)	
2	38 (18.5)	14 (18.4)	4 (14.3)	16 (20.8)	4 (16.7)	
3	94 (45.9)	31 (40.8)	12 (42.9)	43 (55.8%)	8 (33.3)	
4	53 (25.9)	21 (27.6)	7 (25)	15 (19.5)	10 (41.7)	
Unknown	6 (2.9)	4 (5.3)	2 (7.1)	0	0	
N stage, *n* (%)						0.218
0	16 (7.8)	9 (11.8)	0	5 (6.5)	2 (8.3)	
1	91 (44.4)	27 (35.5)	13 (46.4)	41 (53.2)	10 (41.7)	
2	79 (38.5)	32 (42.1)	10 (35.7)	30 (39.0)	7 (29.2)	
Nx	19 (9.3)	8 (10.5)	5 (17.9)	1 (1.3)	5 (20.8)	
Unknown						
M stage, *n* (%)						0.317
M0	142 (69.3)	45 (59.2)	21 (75.0)	58 (75.3)	18 (75.0)	
M1a	39 (19.0)	20 (26.3)	3 (10.7)	12 (15.6)	4 (16.7)	
M1b	7 (3.4)	3 (3.9)	1 (3.6)	3 (3.9)	0	
M1c	17 (8.3)	8 (10.5)	3 (10.7)	4 (5.2)	2 (8.3)	
Unknown	0	0				
Multifocal primary tumours, *n* (%)	63 (30.7)	16 (21.1)	9 (32.1)	33 (42.9)	5 (20.8)	0.049
Lymph node resections						
Median, *n* (range)	8.0 (0–62)	5 (0–30)	6 (0–24)	11 (0–42)	5.5 (0–62)	0.014
Pts. with ≥8 LN, *n* (%)	108 (52.9)	34 (45.3)	13 (46.4)	51 (66.2)	10 (41.7)	0.037
LN+/patient, median (range)	2.0 (0–15)	2.0 (0–15)	2 (0–10)	2 (0–13)	2.5 (0–9)	0.523
Resection margins, *n* (%)						0.716
R0	157 (76.6)	57 (75.0)	20 (71.4)	64 (83.1)	16 (66.7)	
R1	20 (9.8)	8 (10.5)	2 (7.1)	9 (11.7)	1 (4.2)	
R2	26 (12.7)	9 (11.8)	6 (21.4)	4 (5.2)	7 (29.2)	
Unknown	2 (1.0)	2 (2.6)	0	0	0	
Curative surgery, *n* (%)	137 (66.8)	42 (55.3)	20 (71.4)	60 (77.9)	15 (62.5)	0.027

**Table 4:** Multivariate Cox regression analysis of variables associated with overall survival in patients with SI-NETs.

Variable	Univariate		Multivariate	
	HR (95% CI)	*p*-Value	HR (95% CI)	*p*-Value
Tumour resected	0.40 (0.25–0.62)	<0.001	0.52 (0.29–0.91)	0.022
WHO grade 2	1.51 (1.00–2.27)	0.047	1.28 (0.83–1.97)	0.265
ENETS stage	1.53 (1.13–2.07)	0.006	1.81 (1.26–2.61)	0.001
Female sex	1.40 (0.98–2.06)	0.068	1.32 (0.88–1.99)	0.18
Age at diagnosis	1.06 (1.04–1.09)	<0.001	1.07 (1.05–1.10)	0.001
Diagnosis in second period	0.58 (0.37–0.90)	0.015	0.56 (0.35–0.89)	0.014

HR: hazard ratio; CI: confidence interval; ENETS: European Neuroendocrine Tumour Society; WHO: World Health Organization. Diagnosis second period: after 5 November 2012.

**Table 5:** Multivariate Cox regression analysis of variables associated with overall survival in patients with SI-NET that did not undergo R0 resection.

Variable	Multivariate	
	HR (95% CI)	*p*-Value
R1 or R2 resection *	0.64 (0.30–0.1.37)	0.25
WHO grade 2	1.28 (0.60–2.72)	0.53
ENETS stage	2.29 (1.14–4.57)	0.019
Female sex	1.27 (0.61–2.65)	0.52
Age at diagnosis	1.07 (1.03–1.12)	0.002

HR: hazard ratio; CI: confidence interval; * no resection as reference; ENETS: European Neuroendocrine Tumour Society; WHO: World Health Organization.

## Data Availability

The original data cannot be made public available.
